# Comparing urine and stool gluten immunogenic peptides for detecting compliance to gluten-free diets

**DOI:** 10.1038/s41390-025-04266-9

**Published:** 2025-07-24

**Authors:** Konstantinos Gkikas, Laura Gianolio, Miles Kavanagh, Bernadette White, Caroline Kerbiriou, Maria Lima, Vaios Svolos, Richard Hansen, Richard K. Russell, Konstantinos Gerasimidis

**Affiliations:** 1 Human Nutrition, School of Medicine, University of Glasgow, New Lister Building, Glasgow Royal Infirmary, Glasgow, UK; 2 Department of Paediatrics, Vittore Buzzi Children’s Hospital, University of Milan, Milan, Italy; 3 Department of Nutrition & Dietetics, University of Thessaly, Trikala, Greece; 4 Department of Paediatric Gastroenterology, Hepatology and Nutrition, Royal Hospital for Children, Glasgow, UK; 5 Department of Child Health, Division of Respiratory Medicine and Gastroenterology, School of Medicine, University of Dundee, Dundee, UK; 6 Department of Paediatric Gastroenterology, Hepatology and Nutrition, Royal Hospital for Children and Young People, Edinburgh, UK

## Abstract

**Background:**

Detection of gluten immunogenic peptides (GIP) is a potential objective biomarker of adherence to gluten-free diet (GFD). As current methods require specialized laboratories, we evaluated novel point-of-care (POC) kits for GIP detection in stool and urine.

**Methods:**

Ten children with Crohn’s disease followed a 3-week GFD, after 8 weeks of exclusive enteral nutrition (EEN). 78 stool and urine samples were collected; one before EEN completion, six during the GFD, and one after return to unrestricted diet. Single samples were collected from 17 healthy adults after a 7-day EEN. Stool GIP was measured with reference ELISA (S-REF) and POC (S-POC) kits; urine GIP with POC kits (U-POC).

**Results:**

Non-adherence to GFD was detected in 8/10 patients using S-REF, compared to 2/10 patients using conventional dietary assessment. Substantial inter-class agreement was noted between S-REF and S-POC (88% concordance, kappa = 0.74). Lower GIP levels, measured with S-REF, were seen in discordant compared to positive concordant samples. The optimal S-POC detection threshold was 0.11 mg/kg (sensitivity: 100%, specificity: 91%, *p* < 0.001). Agreement between S-REF and U-POC was lower (concordance: 73%, kappa = 0.39).

**Conclusions:**

The S-POC kit is a suitable alternative for GIP detection, demonstrating high sensitivity and specificity except at very low GIP levels. Detection of GIP in urine is less accurate.

**Impact:**

- Detection of gluten immunogenic peptides (GIP) offers a promising objective biomarker for monitoring adherence to gluten-free diets. However, current methods require specialized labs and long processing times.
- By measuring GIP in stool, we identified children with Crohn’s disease who were non-adherent despite standard assessments indicating otherwise.
- A point-of-care GIP kit demonstrated high sensitivity and specificity in detecting GIP in stool making it a potential alternative to the reference ELISA method. In contrast, GIP detection using urine kits was suboptimal.
- Our study supports the potential clinical utility of bedside point-of-care GIP kits that may improve treatment adherence and efficacy of gluten-free-diets.

## Introduction

The cornerstone of the management of celiac disease (CeD) is lifelong adherence to a gluten-free diet (GFD).^1^ Similarly, exclusive enteral nutrition (EEN), which is also gluten-free, is an effective treatment for induction of remission in active Crohn’s disease (CD).^2^ Strict adherence to a GFD in CeD, and to EEN in CD, is of paramount importance for optimal treatment outcomes. Given the inherent limitations of conventional dietary assessment methods, including recall bias and misreporting, it is essential to develop objective adherence biomarkers to differentiate between ineffective treatment and lack of response due to poor adherence.

Whilst the measurement of tissue transglutaminase antibody is highly sensitive and specific in the differential diagnosis of CeD, elevated titers can take up to two years to normalize in patients adhering to a GFD, and its sensitivity in detecting transient dietary transgressions is relatively poor.^3^ The detection of gluten immunogenic peptides (GIP) in stool has been recently suggested as an objective biomarker of adherence to GFD. In a previous study in children with CeD, only 17% of patients who were deemed non-adherent to GFD, based on detection of GIP in stool, were correctly identified by the hospital dietitians.^4^ Thus, stool GIP can potentially be added to routine laboratory tests to improve assessment of adherence to GFD and detect accidental exposure to gluten in patients with CeD not responding to a GFD.^4,5^

In CD, there is a lack of biomarkers for assessment of adherence to EEN, both in research and routine clinical practice. Recently, the Glasgow Exclusive Enteral Nutrition Index of Compliance was developed to objectively assess adherence to EEN using microbial biomarkers and pH in stool.^6^ Measurement of GIP in stool, as a biomarker of adherence to EEN, has also been explored in children with CD undergoing induction therapy with EEN. In patients deemed to fully adhere to EEN by clinical dietetic review, GIP was detected in 23% of stool samples at EEN completion, indicating a lack of strict adherence. Most importantly, the same subgroup of patients showed a poorer response to EEN therapy compared to those with undetectable GIP, as indicated by persistent elevation of fecal calprotectin levels, a biomarker of gut inflammation.^7^

Uncertainties remain for the use of GIP as a biomarker of adherence to GFD. These include the optimal type of biospecimen to test, the amount of ingested gluten needed for GIP detection, and the timeframe within which GIP can be detected in each biospecimen (11). To date, measurement of GIP has primarily been performed in stool using an enzyme-linked immunoassay method (ELISA) which requires specialized equipment, trained personnel, and a considerable turnaround time. Collecting stool samples can also be burdensome for some patients, highlighting the need for suitable alternatives. We aimed to compare the performance of novel GIP detection kits for point-of-care (POC) measurement in stool and urine. These kits were compared against the current reference ELISA method in stool, using samples from patients with CD and healthy volunteers (HV) after following a GFD.

## Methods

### Participants and study design

The present study included samples and data collected from two prospective intervention studies.

### Crohn’s disease cohort

In the first study (IPENS; clinical trials.gov identifier: NCT04225689), children and young adults with CD [female: 3/10 (30%), median (IQR) age: 13.1 (11.9, 15.2)], who had achieved clinical remission (weighted Pediatric CD Activity Index<12.5)^8^ with a 6–8-week course of EEN, were recruited from 11 pediatric hospitals across the UK. Immediately following completion of EEN, patients transitioned to the Crohn’s Disease TReatment with EATing diet (CD-TREAT) diet for 21 days. CD-TREAT is a food-based diet that replicates the nutritional composition of EEN and is therefore gluten-free.^9^ After 21 days on the CD-TREAT diet, patients returned to their unrestricted habitual diet. A pair of stool and urine samples were collected on the same day at eight different timepoints; just before EEN completion and at 3, 6, 9, 12, 15, and 21 days during CD-TREAT. A final sample was collected upon return to unrestricted diet at day 30.

### Healthy volunteer cohort

In the second study (ENIP study; clinicaltrials.gov identifier: NCT0682809), HV [female: 13/17 (76%), median (IQR) age: 30.5 (25.3, 49.3)], were recruited via advertisement from the community in Glasgow to explore the efficacy of EEN and partial enteral nutrition on immune host signatures. We included a subset of participants from this study who consumed EEN as their sole source of nutrition for seven consecutive days. A single pair of stool and urine samples was collected, on the same day, upon completion of the 7-day EEN course.

### Subjective adherence to gluten-free diet

In the CD cohort, adherence to the GFD was assessed against checklists of CD-TREAT food items or meals provided for the entire duration of the intervention (21 days). In HV, adherence to EEN was assessed through face-to-face questioning and prompting (i.e., complied through the full intervention, complied for more than half of the intervention, complied for less than half of the intervention), and by counting empty EEN formula tins at the end of the intervention.

### Sample collection

Participants collected their entire bowel movement and a spot urine sample on the same day. Both stool and urine samples were collected fresh and were immediately stored in −20 °C domestic freezers, provided to participants at home, before being transported frozen to the laboratory. Samples were defrosted at room temperature, homogenized with mechanical kneading, and processed for analysis of GIP.

### Measurement of GIP

Detection of GIP was performed using three different kits. In stool, the IVYLISA GIP Stool kit (S-REF) and the GlutenDetect GIP Stool kit (S-POC) were used. The same sample was used to assess GIP presence using both kits. The S-REF is the most widely used kit to detect GIP in stool, uses ELISA technology, provides quantitative estimation of GIP, and was considered as the reference method in our analysis. In contrast, the S-POC is intended for home testing or point-of-care use, utilizes lateral flow strip technology, and can only provide qualitative results (presence/absence of GIP). The IVYCHECK GIP urine kit (U-POC) which is intended for professional use, also employs lateral flow strip technology. Like the S-REF kit, it can provide quantitative results using an immunochromatographic test strip reader.

The lowest limits of detection were 0.078 mg/kg, 0.3 mg/kg, and 2.5 ng/mL for the S-REF, S-POC, and U-POC, respectively. All products were purchased by Biomedal S. L., Seville, Spain, and all laboratory experiments were performed according to the manufacturer’s instructions.

### Statistical analysis

For all three kits, GIP concentration was classified in a binary manner. Patients with GIP below the limits of detection of the assay were classed as “negative”, whereas those with detectable GIP as “positive”. Cohen kappa statistics were used to assess the inter-class agreement between two groups (e.g., S-REF vs S-POC, S-REF vs U-POC) and Fleiss’s kappa to compare agreement across all three groups. The strength of inter-class agreement between the groups was assessed using the criteria proposed by Landis and Koch.^10^

The S-REF also provides quantitative results. For stool samples with GIP concentration below the assay’s detection limit, where no absolute values could be generated, data were imputed using a value equal to half the assay’s lowest detection limit.

The concentration of GIP, measured with S-REF, was compared between the discordant inter-class classification groups using the Mann–Whitney U test. Correlations between GIP concentration measured with the S-REF and U-POC kits, respectively, were performed using Spearman’s rank test. We used the GIP concentration measured with S-REF as the continuous variable and the presence or absence of GIP detected by S-POC as the classification variable to perform receiver operating characteristic (ROC) analysis. This analysis aimed to identify the optimal cut-off values, using Youden’s J index, above which S-POC and U-POC can best detect GIP in stool and urine, respectively. This analysis was performed using the *ROCR* and *verification* packages on R Studio. All statistical analyses were performed using R studio (Version 4.2.2).

### Ethical approval

Approval to conduct the two original studies was granted by the NHS West of Scotland Research Ethics Committee (West of Scotland REC 5 19/WS/0163 for the IPENS study) and the University of Glasgow MVLS Ethics Committee (REF: 200220086 for the ENIP study). All participants provided signed informed consent.

## Results

### Comparison between GIP kits and dietary assessment methods

In total, 95 pairs of urine and stool samples were collected and analyzed for the detection of GIP. Of these, 78 (between six to eight pairs of stool and urine samples per patient) were collected from 10 children with CD, and 17 pairs of samples were collected from 17 HV.

In the CD cohort, 8/10 (80%) patients reported strict adherence to the GFD, while 2/10 (20%) patients reported the consumption of gluten-containing food in their food checklists. Hence, we expected to detect GIP in at least one stool sample from those two participants reporting non-compliance during the GFD. Unexpectedly, GIP was detected in at least one sample during the GFD period in 8/10 (80%) patients using S-REF, in 7/10 (70%) patients using S-POC, and in 6/10 (60%) patients using U-POC. Collectively, GIP was detected in 28/78 (36%) samples using S-REF, in 18/78 (23%) using S-POC, and in 20/78 (26%) using U-POC (Supplemental Table S1).

In the HV cohort, 12/17 (71%) participants reported strict adherence to GFD, while 4/17 (24%) reported being adherent for more than half the days of the intervention. No dietary assessment data were available for one participant. Interestingly, GIP was detected in 8/17 (47%) samples using S-REF, in 7/17 (41%) using S-POC, and in 6/17 (35%) using U-POC (Supplemental Table S2).

### Comparison between S-REF and S-POC kits

**Fig. 1:**
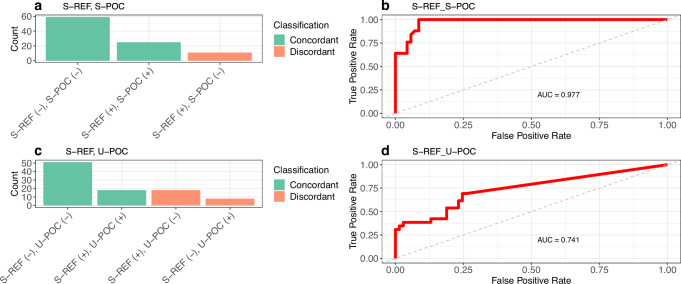
Performance of point-of-care kits for detection of gluten immunogenic peptides in faeces and in urine. **a** Number of samples concordant or discordant for GIP between S-REF (Stool ELISA) and S-POC (Stool POC); **b** ROC curve analysis displaying the predictive ability of S-POC to detect GIP across different concentrations of GIP measured with S-REF; **c** Number of samples concordant or discordant for GIP between S-REF (Stool ELISA) and U-POC (Urine POC); **d** ROC curve analysis displaying the predictive ability of U-POC to detect GIP across different concentrations of GIP measured with S-REF.

Detection of GIP differed between S-REF and S-POC in 11/95 (12%) paired samples [CD: 10/78 (13%); HV: 1/17 (6%)] (Fig. 1a). In all 11 discordant samples, GIP was detected with S-REF but not with S-POC. Notably, all samples that tested positive with S-POC were also positive with S-REF.

When pooling all sample data, the inter-class agreement between the two kits was substantial [Cohen’s κ (SE): 0.74 (0.10)]. A similar level of agreement was observed for the CD cohort [0.70 (0.11)] and an almost perfect agreement was noted between the two kits in the HV cohort [0.88 (0.24)].

Setting S-REF as the reference method, the concentration of GIP was significantly lower in the discordant compared with the positive concordant samples [GIP median (Q1, Q3): 0.13 (0.08, 0.19) vs. 0.45 (0.17, 2.62) mg/kg mg/kg; *p* = 0.001].

Using the concentration of GIP, measured with S-REF, as a continuous variable and the presence/absence of GIP, measured with S-POC, as the classification variable, we performed ROC analysis to determine optimal cut-offs above which S-POC can detect GIP. A ROC curve with an AUC of 0.98 (*p* < 0.001) was generated, identifying an optimal GIP cut-off of 0.11 mg/kg. At this threshold, a sensitivity of 100% and a specificity of 91% was achieved (Fig. 1b).

### Comparison between S-REF and U-POC kits

Detection of GIP differed between S-REF and U-POC in 26/95 (27%) paired samples CD: 20/78 (26%); HV: 6/17 (35%) (Fig. 1c). The most common form of discordance between the two kits was the detection of GIP in stool but not in urine samples [18/26 (69%)]. GIP was detected in urine, but not in stool, in 8/26 (31%) samples.

The inter-class agreement between the two kits was fair-to-moderate [Cohen’s κ (SE); all samples: 0.39 (0.1); CD: 0.41 (0.11); HV: 0.28 (0.24)]. Positive weak-to-moderate correlations were also observed between the concentrations of GIP assayed with S-REF and U-POC in samples from the full cohort (Spearman’s rho = 0.36, *p* < 0.001) and in the CD cohort (Spearman’s rho=0.35, *p* = 0.002). No significant correlations were observed in the samples from HV (*p* = 0.33). In the subset of samples in which GIP was detected using both kits (*n* = 18 samples for both CD and HV), no significant correlation was observed between the two kits (*p* = 0.8).

The concentration of GIP, measured using S-REF, did not significantly differ between concordant and discordant positive samples [GIP median (Q1, Q3) values: 0.49 mg/kg (0.13, 3.14) vs 0.19 mg/kg (0.13, 0.41), *p* = 0.16].

A ROC curve with an AUC of 0.78 (*p* < 0.001) was generated using GIP concentration measured with S-REF as the continuous variable and the detection of GIP using U-POC as the classification variable. The optimal concentration for detection of GIP with the urine kit was 0.08 mg/kg, yielding a modest sensitivity of 69% and a specificity of 75% (Fig. 1d).

### Comparison of all three kits (S-REF vs S-POC vs U-POC)

**Fig. 2:**
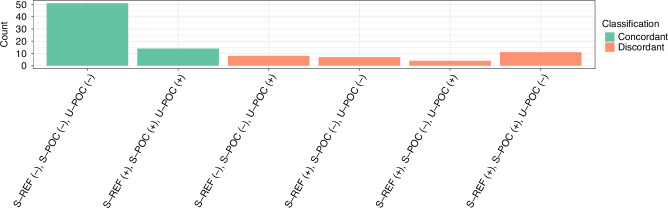
Number of samples concordant or discordant for GIP between S-REF (Stool ELISA), S-POC (Stool POC) and U-POC (Urine POC).

When we combined data from all three different kits, concordance was observed in 65/95 (68%) triads of samples, of which 14 triads were positive for GIP and 51 were negative (Fig. 2). Discordance was observed in 23/78 (29%) samples from patients with CD and in 7/17 samples (41%) from HV. Moderate agreement was observed among all three kits (Fleiss’ κ (SE)); all patients: 0.50 (0.06); CD: 0.51 (0.07); HV: 0.43 (0.14).

The most common form of discordance between the three kits was the detection of GIP in stool using both S-REF and S-POC, but not in urine using U-POC [11/30] (37%) (Fig. 2). This was followed by the detection of GIP in urine, but not in stool, using either S-REF or S-POC in 8/30 (27%) samples. In 7/30 (23%) samples, GIP was detected only with S-REF but not with S-POC or U-POC.

**Fig. 3:**
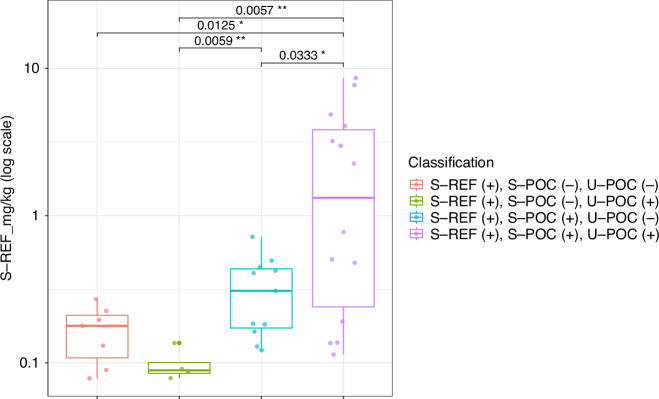
Comparison of GIP concentration in samples positive for GIP detection using the S-REF kit across discordant classifications. Y axis displays log-transformed scale for visualization purposes.

Next, we investigated the reasons for the observed discordance between the three different kits by comparing the concentration of GIP, measured with S-REF, across the various inter-class classifications. The highest concentration of GIP was observed in samples where GIP was detected with all kits [median (Q1, Q3): 1.52 (0.18, 4.26) mg/kg]. In contrast, the lowest GIP concentration was observed in samples where GIP was detected using S-REF and U-POC, but not with S-POC. Higher GIP concentrations were detected in samples that were GIP positive using both S-REF and S-POC, but not with U-POC, compared to samples that were GIP positive with S-REF and U-POC, but not with S-POC (*p* = 0.011, Fig. 3).

Interestingly, in the two patients with CD who strictly adhered to the GFD, as shown by the absence of GIP detection in any of the stool samples collected from day three to day 21 using S-REF (Supplemental Table S1), no GIP was detected using either U-POC or S-POC. Perfect concordance, defined as detection of GIP by all three kits, was observed in 7/9 (78%) samples from patients with CD, 10 days after returning to a gluten-containing diet. In the 2/9 (22%) discordant samples, GIP concentrations, measured with S-REF, were 0.0895 mg/kg and 0.0871 mg/kg, respectively, both close to the detection limit of S-REF (0.078 mg/kg). In contrast, in the 7/9 (78%) concordant samples, GIP concentration was higher than the upper limit of the dynamic range of S-REF (1.25 mg/kg).

## Discussion

To our knowledge, this is the first study to assess the performance of two POC kits for detecting GIP in stool and urine samples, particularly in longitudinal samples collected from the same participants exposed to GFD. Collectively, our analysis revealed substantial agreement between the two stool kits. This indicates the potential of bedside, point-of-care use for the S-POC kit, although its performance was slightly inferior for samples with very low concentrations of GIP. In contrast, the performance of U-POC was poor, challenging its clinical utility. By using an objective, highly sensitive, and specific biomarker of gluten exposure, we identified patients who were not fully adherent to a GFD but who would otherwise have been missed by standard dietetic assessment. These findings align with previous research showing that urine and stool GIP were detected in 20% of patients considered strictly adherent to a GFD using dietetic assessment.^11^ This further highlights the need to develop and use objective markers of adherence in routine clinical practice.

Previous studies have demonstrated the low sensitivity of U-POC in detecting GIP in urine samples from patients with CeD consuming low levels of gluten.^12,13^ A recent study found that whilst S-REF detected GIP in at least one stool sample from 40/52 (77%) patients deemed compliant to a GFD for over 12 months based on standard dietetic assessment, none of the paired urine samples had detectable GIP measured with U-POC.^14^ In accordance, the present study showed substantial discordance (27%) in GIP detection rates between S-REF and U-POC.

Several possible scenarios can explain the discrepancies in GIP detection rates between S-REF and U-POC. Low gluten intake could explain the absence of GIP detection in urine samples. However, in our analysis, we did not observe significant differences in median GIP concentration, measured with S-REF, between concordant positive and discordant samples. Another explanation could be the timing of gluten ingestion. Whilst the optimal detection time of GIP in urine is six to nine hours following gluten ingestion, GIP in stool is typically detected within one to two days following gluten ingestion and, depending on inter-individual variations in intestinal transit time, can remain present in stool for up to seven days.^15^ Testing multiple urine samples rather than relying on spot samples may improve the performance of U-POC. However, such approaches may become cumbersome, impractical, and expensive.^16^

In contrast to U-POC, the performance of S-POC has not been studied extensively. Costa et al. showed 91% concordance in GIP detection rates between S-REF and S-POC kits in samples from patients with CeD.^15^ In agreement, we showed concordance in 89% of all samples assayed. Notably, the optimal threshold identified in our ROC analysis (0.11 mg/kg), above which the sensitivity of S-POC was 100%, is only marginally higher than the detection limit of S-REF (0.078 mg/kg). Whilst it is difficult to accurately estimate gluten intake using GIP concentrations in stool, oral gluten challenge studies by Syage et al. showed that stool GIP levels of 0.11 mg/kg correspond to an estimated daily gluten intake of approximately 116 mg,^17^ or 1/15^th^ of a 30 g slice of wheat bread.^18^ This demonstrates that S-POC is sensitive enough to detect small gluten exposures but might be unable to detect cases of accidental exposure or consumption of traces of gluten. Although no established threshold for safe gluten intake exists for patients with CeD, intakes as low as 10-50 mg/day may trigger symptoms.^19^ The potential impact of other real-world factors, such as improper sample handling or collection or assessing GIP detection in different biospecimens, were minimized in our study by using the same stool sample for both S-REF and S-POC.

**Fig. 4:**
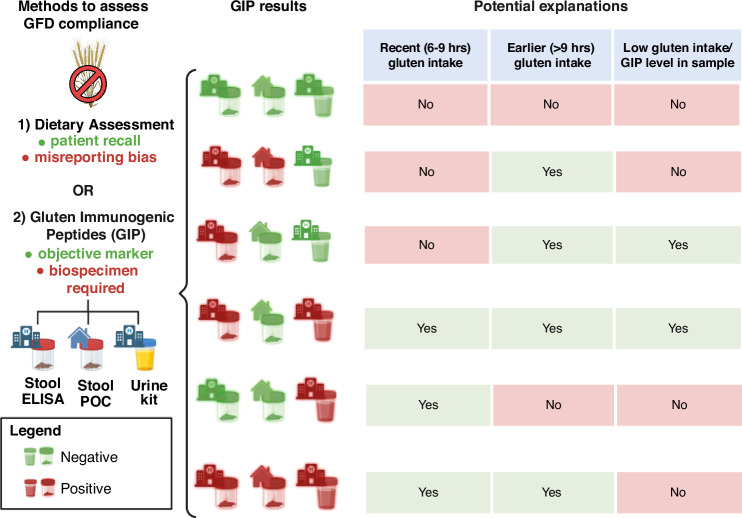
Potential explanations for variability in GIP detection between the S-REF (Stool ELISA), S-POC (Stool POC) and U-POC (Urine POC) kits along with potential implications for clinical practice in assessing compliance to gluten-free diets (GFD); green icon indicates lack of GIP detection; red indicates GIP detection.

Our analysis showed that strict adherence to a GFD can be confirmed using all three GIP detection kits. Similarly, consistent exposure to gluten, particularly at high levels, can be identified, in most cases, using all three kits. While we are unable to provide definitive reasons explaining the various forms of discordance observed among the three different kits, we offer potential explanations in Fig. 4. It is worth mentioning that active CD or CeD with overt clinical symptoms (e.g., lack of adherence to GFD) may influence intestinal transit time; hence influencing the timeframe within GIP can be detected. Whilst the effect of intestinal transit time on GIP detection has not been directly assessed, a recent study found no association between bowel movement frequency and timing of GIP detection in patients with CeD.^14^ Notably, in our study, S-POC performed similarly in patients with CD and HV and overall results were largely consistent, suggesting that disease status does not significantly impact the performance of the kits.

Aside from performance metrics, other factors such as cost, and ease of use must also be considered. The S-POC does not require additional laboratory resources or specialist personnel and equipment, hence heavily reducing the overall analytical cost. In addition, it is a quick and easy-to-use kit and can be used at home or at point of care.

Our study is limited by the modest sample size and age differences between pediatric patients and healthy adult volunteers that prevented direct comparison. However, the use of multiple, consecutive samples offered us a unique opportunity to explore consistency in the performance of the different kits. Another limitation is the use of spot urine samples and the lack of recording of the timing of gluten consumption prior to sample collection. Nonetheless, all paired samples were collected on the same day.

Whilst the role of GIP testing in clinical practice is currently unclear, a better understanding of the performance of the various kits and in different sample types will provide useful insights into possible utility. The obvious indication is the use of GIP testing to detect patients with reduced adherence to GFD, who would otherwise have been missed by standard dietetic assessment. This can lead to the provision of more comprehensive advice and possibly increase treatment adherence and efficacy.

In conclusion, this study suggests that a POC kit can be a suitable alternative to the reference ELISA method in detecting GIP in stool, showing high performance, particularly in medium to high levels of gluten exposure. While urine collection might be more practical than that of stool, the detection of GIP using urine kits was suboptimal and their utility therefore in clinical practice might be limited.

## Supplementary information

Supplementary Tables

## Data Availability

Anonymized data may become available to third parties after request to the corresponding author and only for those patients who provided written consent for this specific aspect of study participation.
