# Supplementary material for: Comparing urine and stool gluten immunogenic peptides for detecting compliance to gluten-free diets
Source: Pediatr Res. 2025 Jul 24;99(3):1181–6. doi: 10.1038/s41390-025-04266-9 (PMC13021495; doi:10.1038/s41390-025-04266-9)
Supplement: Supplementary file 1 — Supplementary Tables [file 41390_2025_4266_MOESM1_ESM.pdf]

Supplementary Tables

Supplemental Table S1 Detection of GIP in consecutive paired stool and urine samples in patients with CD, using the three different kits (S-REF, S-POC, U-POC)

	EEN end			Day 3			Day 6			Day 9			Day 12			Day 15			Day 21			Day 30		
	S-REF	S-POC	U-POC	S-REF	S-POC	U-POC	S-REF	S-POC	U-POC	S-REF	S-POC	U-POC	S-REF	S-POC	U-POC	S-REF	S-POC	U-POC	S-REF	S-POC	U-POC	S-REF	S-POC	U-POC
CD_01	-	-	-	-	-	-	+	+	-	+	+	-	+	+	+	-	-	-						
CD_02	-	-	-	-	-	-	-	-	-	-	-	-	+	-	-	+	-	-	+	+	+	+	-	-
CD_03	-	-	-	-	-	-	-	-	-	-	-	-	-	-	-	-	-	-	-	-	-	+	+	+
CD_04	-	-	+	+	-	+	-	-	+	-	-	-	-	-	-	-	-	+	-	-	-	+	-	+
CD_05	-	-	-	-	-	+	-	-	-	+	+	-	+	+	-	-	-	-	+	+	-	+	+	+
CD_06	-	-	-	-	-	-	-	-	-	+	+	-	-	-	-	-	-	-	-	-	-	+	+	+
CD_07	-	-	+	-	-	-	-	-	-	-	-	-	-	-	-	-	-	-	+	+	+	+	+	+
CD_08	-	-	-	-	-	-	-	-	-	-	-	-	-	-	-	-	-	-	-	-	-	+	+	+
CD_09	-	-	-	-	-	-	+	-	-	+	-	-	+	-	-	+	-	-	+	+	-	+	+	+
CD_10	-	-	-	-	-	-	-	-	-	+	-	+	-	-	-	+	+	+	-	-	+	+	+	+

(+) indicates GIP detection, (-) indicates no GIP detection

CD: Crohn’s disease; EEN: exclusive enteral nutrition; S-REF: Stool ELISA kit; S-POC: stool point-of-care kit; U-POC: urine point-of-care kit

Supplemental Table S2 Detection of GIP in spot paired stool and urine samples in healthy volunteers, using the three different kits (S-REF, S-POC, U-POC)

	S-REF	S-POC	U-POC
HV_01	-	-	-
HV_02	-	-	+
HV_03	+	+	-
HV_04	+	+	-
HV_05	-	-	-
HV_06	+	+	-
HV_07	-	-	-
HV_08	-	-	-
HV_09	+	+	+
HV_10	+	+	+
HV_11	+	-	+
HV_12	-	-	-
HV_13	+	+	-
HV_14	-	-	-
HV_15	-	-	-
HV_16	-	-	+
HV_17	+	+	+

(+) indicates GIP detection, (-) indicates no GIP detection
HV: healthy volunteers S-REF: Stool ELISA kit; S-POC: stool point-of-care kit; U-POC: urine point-of-care kit
